# Impact of anemia requiring transfusion or erythropoiesis-stimulating agents on new-onset cardiovascular events and mortality after continuous renal replacement therapy

**DOI:** 10.1038/s41598-024-56772-1

**Published:** 2024-03-19

**Authors:** Junseok Jeon, Danbee Kang, Hyejeong Park, Kyungho Lee, Jung Eun Lee, Wooseong Huh, Juhee Cho, Hye Ryoun Jang

**Affiliations:** 1 Division of Nephrology, Department of Medicine, Samsung Medical Center, Sungkyunkwan University School of Medicine, 81 Irwon-ro, Gangnam-gu, Seoul, 06531 Republic of Korea; 2 Center for Clinical Epidemiology, Samsung Medical Center, Sungkyunkwan University School of Medicine, 81 Irwon-ro, Gangnam-gu, Seoul, 06531 Republic of Korea

**Keywords:** Critical illness, Anemia, Continuous renal replacement therapy, Cardiovascular events, Mortality, Kidney diseases, Risk factors, Anaemia, Renal replacement therapy

## Abstract

Anemia is common in critically ill patients undergoing continuous renal replacement therapy (CRRT). We investigated the impact of anemia requiring red blood cell (RBC) transfusion or erythropoiesis-stimulating agents (ESAs) on patient outcomes after hospital discharge in critically ill patients with acute kidney injury (AKI) requiring CRRT. In this retrospective cohort study using the Health Insurance Review and Assessment database of South Korea, 10,923 adult patients who received CRRT for 3 days or more between 2010 and 2019 and discharged alive were included. Anemia was defined as the need for RBC transfusion or ESAs. Outcomes included cardiovascular events (CVEs) and all-cause mortality after discharge. The anemia group showed a tendency to be older with more females and had more comorbidities compared to the control group. Anemia was not associated with an increased risk of CVEs (adjusted hazard ratio [aHR]: 1.05; 95% confidence interval [CI]: 0.85–1.29), but was associated with an increased risk of all-cause mortality (aHR: 1.41; 95% CI 1.30–1.53). For critically ill patients with AKI requiring CRRT, anemia, defined as requirement for RBC transfusion or ESAs, may increase the long-term risk of all-cause mortality.

## Introduction

Anemia is a very common condition in critically ill patients, and approximately 30–50% require a red blood cell (RBC) transfusion^1,2^. Various factors can contribute to anemia in critically ill patients, including major bleeding, significant hemolysis, insufficient erythropoietin (EPO) production, impaired bone marrow response to EPO, nutritional deficiencies, obscure blood loss from frequent blood sampling, occult gastrointestinal bleeding, or invasive procedures^3–6^. Inflammatory cytokines can disrupt iron homeostasis, hinder EPO production, and suppress bone marrow erythropoiesis^7–9^.

EPO is an essential hormone for RBC production, primarily secreted by renal interstitial fibroblasts^10,11^. Kidney dysfunction can inevitably aggravate insufficient production of EPO^12^. Therefore, critically ill patients with acute kidney injury (AKI) are at a higher risk of developing anemia due to both kidney dysfunction and the severity of their illness. Renal replacement therapy, including continuous renal replacement therapy (CRRT), can further exacerbate anemia because of blood loss from hemofilter clotting that cannot be returned^13^ as well as bleeding associated with catheters or anticoagulation^14^.

Anemia has been associated with poor outcomes in various clinical settings^15–17^. AKI is recognized as a long-term risk factor for cardiovascular events and mortality^18^. One study demonstrated that the substantial impact of AKI on long-term outcomes was, in part, attributed to the development of anemia following AKI^19^. However, the influence of anemia on long-term outcomes in patients with AKI requiring CRRT remains unclear. Therefore, we investigated the impact of anemia during hospitalization on cardiovascular outcomes and all-cause mortality following hospital discharge among critically ill patients with AKI requiring CRRT.

## Results

### Patient characteristics

Of 10,923 patients who survived until hospital discharge, 78% (N = 8,495) had anemia during their hospitalization (RBC transfusion in 5,948, erythropoiesis- stimulating agent [ESA] in 270, and RBC transfusion and ESA in 2,227 patients). The anemia group exhibited a higher average age, greater number of comorbidities (except for diabetes mellitus), higher proportion of septic shock, increased mechanical ventilation usage, and a longer stay in the intensive care unit (ICU) compared to the control group (Table 1).

**Table 1:** Characteristics of the patients.

	Overall	Control	Anemia	*P* value
	(N = 10,923)	(N = 2428)	(N = 8495)	
Age, years	63.4 ± 16.2	59.7 ± 16.8	64.4 ± 15.8	< 0.001
Sex, male	6567 (60.1)	1778 (73.2)	4789 (56.4)	< 0.001
Tertiary	5658 (51.8)	1114 (45.9)	4544 (53.5)	< 0.001
Comorbidities				
Charlson index	1.9 (1.9)	1.2 (1.6)	2.0 (1.9)	< 0.001
Chronic liver disease	1372 (12.6)	281 (11.6)	1091 (12.8)	0.1
Diabetes Mellitus	3337 (30.6)	800 (32.9)	2537 (29.9)	0.004
Chronic Kidney Disease	2646 (24.2)	246 (10.1)	2400 (28.3)	< 0.001
Cancer	1554 (14.2)	166 (6.8)	1388 (16.3)	< 0.001
Hypertension	2598 (23.8)	557 (22.9)	2041 (24.0)	0.27
Septic shock	5758 (52.7)	961 (39.6)	4797 (56.5)	< 0.001
Treatment				
CRRT duration, days	6.0 ± 4.2	4.2 ± 1.5	6.5 ± 4.5	< 0.001
Mechanical ventilation	4718 (43.2)	775 (31.9)	3943 (46.4)	< 0.001

Continuous variables and categorical variables are presented as means ± standard deviations and numbers (percentages), respectively.

Charlson comorbidity was defined as the presence of disease within 1 year prior to admission.

*CRRT* continuous renal replacement therapy, *SD* standard deviation.

### Cardiovascular events after hospital discharge

**Figure 1:**
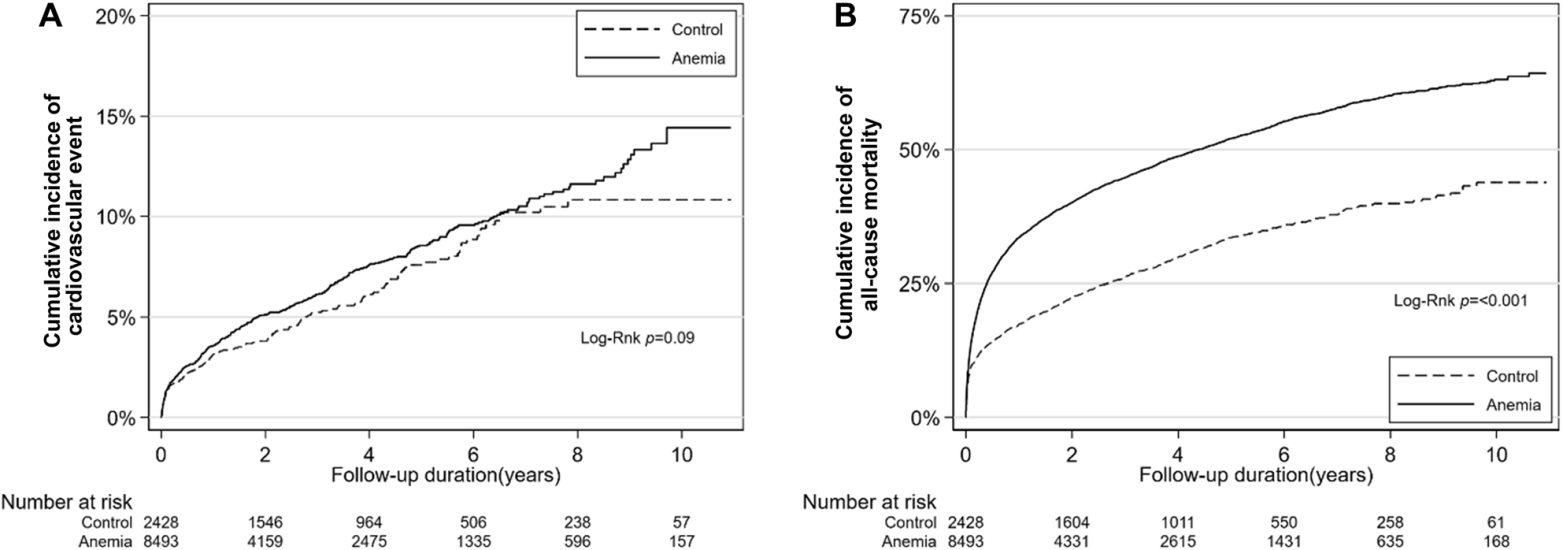
Cardiovascular events and all-cause mortality according to anemia. (**A**) Cardiovascular events and (**B**) all-cause mortality. A cardiovascular event was defined as a composite outcome of heart failure, acute myocardial infarction, revascularization, or stroke.

There were 622 cardiovascular events, of which 141 occurred in the control group and 481 in the anemia group (control group vs. anemia group; 1.6 vs. 2.0 per 100 person-years) (Table 2 and Fig. 1A). Multivariable analysis revealed that the risk of cardiovascular events in the control and anemia groups was similar (adjusted HR: 1.05; 95% CI 0.85–1.29). Stroke was the most frequent type of cardiovascular event in both control and anemia groups (1.0 vs. 1.2 per 100 person-years, respectively). However, the difference between the two groups was not statistically significant (adjusted HR: 0.97; 95% CI 0.75–1.27). Risks of other cardiovascular events, including heart failure, acute myocardial infarction (MI), and revascularization were also comparable between the control and anemia groups.

**Table 2:** Anemia and the risk of cardiovascular events or all-cause mortality.

	Control (N = 2428)	Anemia (N = 8495)	Crude HR (95% CI)	Adjusted* HR (95% CI)
	No. of events (% per patient-year)			
Cardiovascular events^†^	141 (1.6)	481 (2.0)	1.17 (0.98–1.41)	1.05 (0.85–1.29)
Heart failure	35 (0.4)	109 (0.4)	1.24 (0.89–1.71)	1.08 (0.72–1.62)
Acute myocardial infarction	14 (0.2)	59 (0.2)	1.47 (0.86–2.49)	1.50 (0.86–2.62)
Revascularization	25 (0.3)	83 (0.3)	1.18 (0.78–1.77)	1.36 (0.89–2.08)
Stroke	87 (1.0)	285 (1.2)	1.12 (0.88–1.43)	0.97 (0.75–1.27)
All-cause mortality	771 (8.4)	4,323 (17.3)	1.87 (1.73–2.02)	1.41 (1.30–1.53)

*Adjusted for age, sex, tertiary hospital, comorbidities, septic shock, CRRT duration, and mechanical ventilation.

^†^A composite outcome of heart failure, acute myocardial infarction, revascularization, or stroke.

*CI* confidence interval, *HR* hazard ratio.

### All-cause mortality after hospital discharge

During the follow-up, 5,094 deaths occurred after hospital discharge. The anemia group exhibited a significantly higher risk of all-cause mortality compared to the control group (control group vs. anemia group; 8.4 vs. 17.3 per 100 person-years; adjusted HR: 1.41; 95% CI 1.30–1.53; Table 2 and Fig. 1B).

### Subgroup analysis

**Figure 2:**
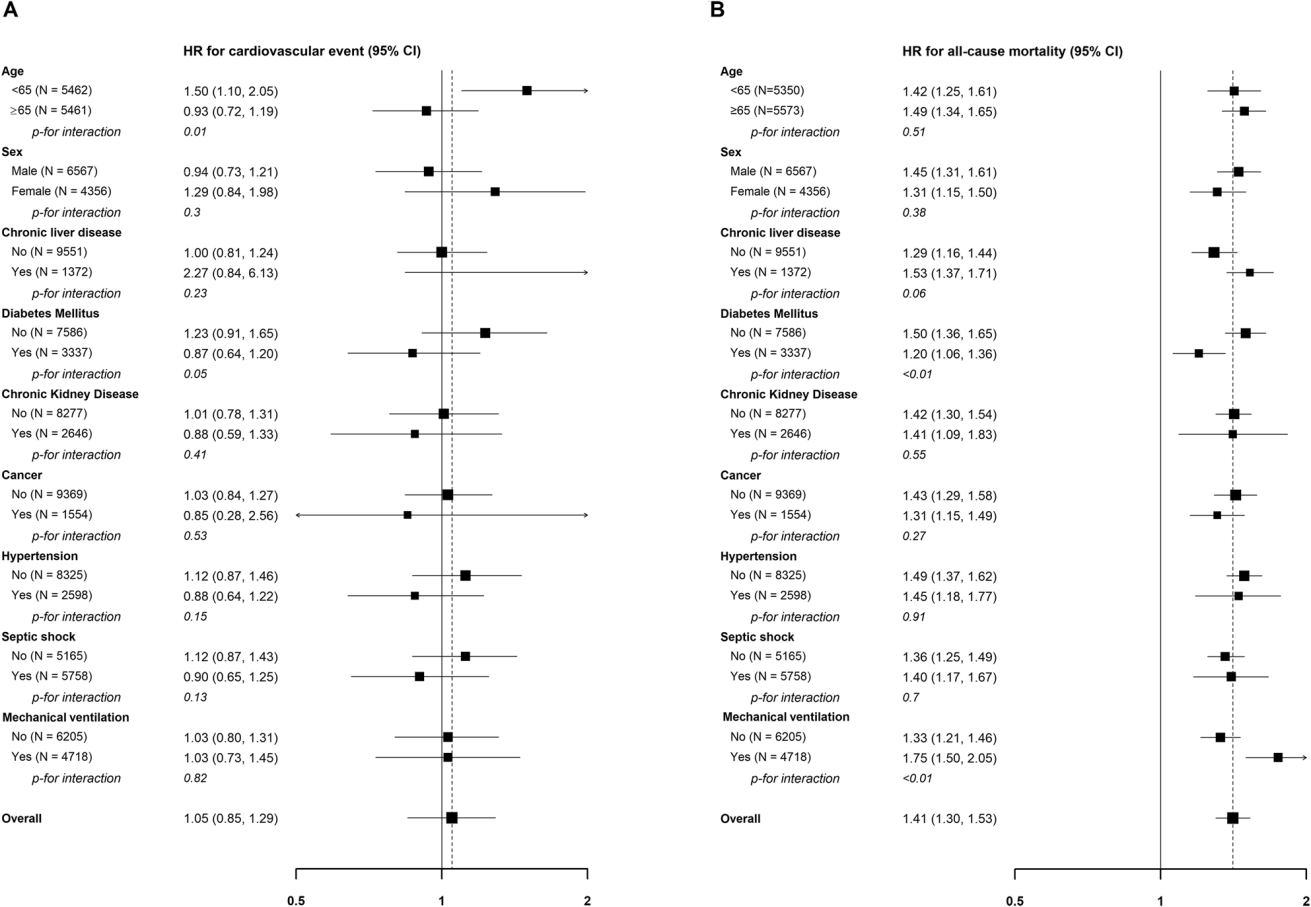
Subgroup analysis stratified according to age, sex, and comorbidities for cardiovascular events and all-cause mortality. (**A**) Cardiovascular events and (**B**) all-cause mortality. A cardiovascular event was defined as a composite outcome of heart failure, acute myocardial infarction, revascularization, or stroke.

The association between anemia and cardiovascular events was not statistically significant in various subgroups, except for age (Fig. 2A). In patients aged ≥ 65 years, there was no difference in cardiovascular events between the control and anemia groups. However, among patients aged < 65 years, the risk of cardiovascular events was significantly higher in the anemia group compared to that in the control group (adjusted HR: 1.50; 95% CI 1.10–2.05). Patient characteristics between the control and anemia groups were comparable in both subgroups, for those aged ≥ 65 years and those < 65 years (Supplementary Table 1).

The association between anemia and an increased risk of all-cause mortality remained consistently significant in all subgroups, including patients with cancer (adjusted HR: 1.40; 95% CI 1.17–1.67 and 1.36) and without cancer (adjusted HR: 1.40; 95% CI 1.25–1.49) (Fig. 2B).

### Sensitivity analysis

In the additional analyses conducted according to the definition of anemia, no significant association was observed between anemia defined by RBC transfusion and the risk of cardiovascular events. However, anemia defined by ESA prescription showed a tendency toward an increased risk of cardiovascular events (adjusted HR: 1.23; 95% CI 0.96–1.57).

Anemia defined by RBC transfusion alone (adjusted HR: 1.53; 95% CI 1.40–1.66) and combination of RBC transfusion and ESA prescription (adjusted HR: 1.23; 95% CI 1.13–1.34) also showed an increased risk of all-cause mortality (Table 3). In contrast, anemia defined by ESA alone was associated with a decreased risk of all-cause mortality (adjusted HR: 0.78; 95% CI 0.64–0.97).

**Table 3:** Cardiovascular events and all-cause mortality according to RBC transfusion and ESA therapy.

	No. patients	No. of events (% per patient-year)	Crude HR (95% CI)	Adjusted* HR (95% CI)
Cardiovascular events^†^				
Control	2428	141 (1.6)	Reference	Reference
RBC transfusion	5948	309 (1.8)	1.08 (0.88–1.32)	1.00 (0.80–1.24)
ESA	270	157 (2.6)	1.46 (1.16–1.83)	1.23 (0.96–1.57)
RBC transfusion and ESA	2277	15 (1.7)	1.01 (0.61–1.65)	0.88 (0.54–1.42)
All-cause mortality				
Control	2428	771 (8.4)	Reference	Reference
RBC transfusion	5948	3107 (17.5)	1.93 (1.78–2.10)	1.53 (1.40–1.66)
ESA	270	83 (9.2)	0.99 (0.80–1.21)	0.78 (0.64–0.97)
RBC transfusion and ESA	2277	1133 (17.7)	1.83 (1.67–2.00)	1.23 (1.13–1.34)

*Adjusted for age, sex, tertiary hospital, comorbidities, septic shock, CRRT duration, and mechanical ventilation.

^†^A composite of heart failure, acute myocardial infarction, revascularization, or stroke.

*CI* confidence interval, *ESA* erythropoiesis-stimulating agent, *HR* hazard ratio, *RBC* red blood cell.

## Discussion

In this large nationwide population-based cohort study, we demonstrated the association between anemia defined as requirement for RBC transfusion or ESA therapy, during hospitalization and an increased risk of post-discharge all-cause mortality in critically ill patients with AKI requiring CRRT. This increased mortality associated with anemia may not be attributable to an increase in cardiovascular events among the older population. However, among individuals < 65 years old, anemia during hospitalization was associated with an increased risk of post-discharge cardiovascular events. Our findings indicate that anemia can serve as a long-term prognostic factor in critically ill patients who have undergone CRRT.

Recent studies have indicated that anemia can persist for a significant period following critical illness^20,21^. In a large cohort study, the one-year recovery rates for moderate anemia (hemoglobin [Hb] < 10.0 g/dL) and severe anemia (Hb < 8.0 g/dL) at hospital discharge were merely 39% and 24%, respectively. Moreover, a higher Hb at hospital discharge was associated with decreased mortality in critically ill patients^20^. Anemia persisting post-critical illness may indicate incomplete recovery from an underlying disease or chronic inflammation^21^. A new syndrome called “persistent inflammation-immunosuppression and catabolism syndrome” (PICS) was proposed to explain the underlying pathological consequences for the high morbidity and mortality observed in patients with chronic critical illness following sepsis or severe injury^22^. The primary mechanism of PICS involves the expansion of a heterogeneous population of inducible immature myeloid cells during critical illness, which predominantly exhibit immunosuppressive and inflammatory properties^22,23^. Therefore, anemia may serve as an indicator for not only the severity of acute illness but also long-term outcomes affected by PICS.

Our study underscores the significance of anemia requiring RBC transfusion during hospitalization as a prognostic factor for long-term mortality after discharge in critically ill patients undergoing CRRT. This aligns with numerous studies that have reported associations between anemia and poor clinical outcomes^24–26^. Notably, there is a lack of evidence supporting the notion that correcting anemia with transfusions leads to improved patient outcomes; rather, transfusions can expose patients to various transfusion-related adverse events^27^. Therefore, restrictive RBC transfusion strategies have been recommended.^28^ Nevertheless, the association between anemia and long-term outcomes in critically ill patients has not been fully elucidated through large-scale cohort studies. A recent study showed that anemia (Hb < 10.0 g/dL) in the first week was associated with long-term morality among critically ill patients^29^, and another study reported that anemia following AKI was associated with long-term adverse outcomes^19^. We analyzed the association between severe anemia and long-term outcomes focusing on severe AKI patients requiring CRRT in a much larger cohort compared to these previous studies. The National Health Insurance Service (NHIS) database did not allow us to determine when CRRT was applied or when blood transfusions or ESAs were administered during hospitalization. Therefore, comparing results over a short period of time can be unreliable. The cumulative incidence of mortality shows that the difference between the control and anemia groups occurs primarily within 1–2 years. Therefore, despite the lack of consistency at the start of follow-up, we believe that the difference in mortality between the control and anemia groups may be related to anemia during hospitalization rather than to new events after discharge. Our findings strongly suggest the importance of vigilant monitoring and management of ongoing anemia and correctable conditions, even after hospital discharge, particularly for critically ill patients with AKI severe enough to require CRRT.

The increase in new-onset cardiovascular events was evident among patients aged < 65 years in the anemia group compared to the control group. However, for patients aged ≥ 65 years, the incidence of cardiovascular events remained comparable regardless of anemia. In contrast, there were no associations between anemia and cardiovascular events according to sex and comorbidities. This age-dependent difference remains unclear. However, a possible explanation is that older patients at a higher risk of cardiovascular events may have been preemptively excluded from our cohort due to their preexisting cardiovascular diseases. Anemia is recognized as a risk factor for cardiovascular diseases, although the causal relationship remains controversial.^15^ Anemia may cause or aggravate cardiovascular diseases through hemodynamic effects^30^ and other mechanisms, such as decreased adiponectin levels and peripheral endothelial progenitor cells.^31,32^ While our study did not demonstrate that severe anemia during hospitalization increased the overall risk of cardiovascular events in critically ill patients, it did reveal the increased risk in patients aged < 65 years. Further research is needed to clarify the causal relationship between anemia and the risk of cardiovascular events according to age.

In our study, we observed that RBC transfusion during hospitalization was associated with an increased risk of all-cause mortality, while ESA alone was associated with a decreased risk of all-cause mortality. Previous studies have revealed inconsistent results regarding the effects of ESA therapy on mortality in critically ill patients, although a recent meta-analysis suggested that ESA therapy may reduce mortality in critically ill patients^33–35^. Moreover, ESA therapy appeared to reduce the requirement for RBC transfusions in critically ill patients, although the clinical relevance is yet to be determined^36^. Although recent guideline suggested not using ESAs to prevent RBC transfusion (conditional recommendation, low certainty)^37^, its use is still controversial, especially in critically ill patients with renal impairment; thus, the application and method of ESA therapy are highly dependent on the preferences of the physician. Since the results of observational studies are inevitably subject to selection bias and confounders, further prospective studies are required to elucidate the impact of ESA therapy on mortality or requirement of RBC transfusion in critically ill patients with AKI.

We aimed to evaluate the effect of anemia on long-term outcomes by assuming that patients receiving RBC transfusion or ESAs without history of surgery or intervention for bleeding control are likely to have significant anemia. However, our study could not distinguish whether adverse outcomes were related to anemia or RBC transfusion per se. Beyond short-term adverse effects, transfusions can also exert long-term effects by mechanisms such as transfusion-related immunomodulation or iron overload^38,39^. Furthermore, white blood cells are known to release several pro-inflammatory mediators during storage^40^. Previous studies reported the association of transfusions with long-term adverse outcomes in various clinical settings such as surgery or myocardial infarction^41–43^. Therefore, further studies that comprehensively evaluate long-term outcomes according to hemoglobin level as well as transfusion are required.”

Our study has several limitations. First, the causal relationship between anemia and mortality or cardiovascular outcomes was not fully elucidated due to the retrospective nature of the study. Additionally, the database of KNIHS had limitations in providing a full comprehensive picture of patient conditions, especially the diagnoses at the time of ICU admission and detailed laboratory findings. Although kidney function plays an important role in the development or persistence of anemia, serum creatinine or cystatin C was not available in this study. Second, as mentioned earlier, the definition of anemia was determined according to RBC transfusion or ESA administration because hemoglobin or hematocrit levels were not available in the KNIHS database. Although a restrictive strategy of RBC transfusion has been widely accepted in clinical practice^28^, the strategy for RBC transfusion and ESA administration can vary among physicians and may be influenced by underlying diseases or personal convictions, including religious considerations. However, we consider that RBC transfusion can reflect anemia in general. Third, the data regarding the persistence or degree of anemia after discharge could not be collected. Fourth, our study focused on patients with AKI who were critically ill enough to require CRRT to reduce heterogeneity in study population, so that the conclusion of our study may be insufficient for generalization to all patients with AKI. Despite these limitations, our study holds clinical significance as it reveals the association between anemia and long-term outcomes in a large cohort of critically ill patients with severe AKI requiring CRRT.

In conclusion, our study demonstrates that anemia, defined as requirement for RBC transfusion or ESA therapy, during hospitalization was associated with an increased risk of post-discharge all-cause mortality in critically ill patients with AKI requiring CRRT. Additionally, the risk of new-onset cardiovascular events was also increased in the patients aged < 65 years in this cohort. Therefore, it is crucial to provide vigilant monitoring and treatment for all correctable conditions, including anemia, to critically ill patients with severe anemia and AKI requiring CRRT, even after hospital discharge. Appropriate ESA therapy with adequate nutritional support including iron supplementation and active rehabilitation after discharge should be considered in patients with persistent anemia and renal impairment.

## Materials and methods

### Study population and design

This retrospective cohort study obtained data from the Korean National Health Insurance Service (KNHIS) database, which provides comprehensive coverage to approximately 97% of the Korean population. The remaining 3% of individuals who are unable to afford national insurance are covered by the Medical Aid Program^44^. Therefore, the KNHIS database effectively represents the entirety of the South Korean population and contains the national records of all covered inpatient and outpatient visits, prescriptions, and procedures. The KNHIS data includes modules on insurance eligibility and medical treatment. The insurance eligibility module contains the information on age, sex, residential area, and income level. The medical treatment module contains information related to treatments, including conditions and prescriptions^45^. This study was conducted with the approval of the KNHIS official review committee (protocol number: NHIS-2022-1-240), and access to the data was granted per approval.

We aimed to evaluate the long-term consequences of anemia in relation to cardiovascular events and mortality after hospital discharge. For this, we included adult patients > 18 years who were admitted to the ICU and received CRRT for ≥ 3 days between January 1, 2010 and December 31, 2019 (N = 119,421). We excluded participants with preexisting end-stage kidney disease (ESKD) (N = 19,605); history of acute MI, stroke, heart failure, or revascularization (N = 43,039); and history of other cardiovascular diseases (N = 28,479). We further excluded patients who received major surgery, endoscopic hemostasis, or vascular embolization (N = 52,946) and those who died during hospitalization (N = 19,294). Thus, a total of 10,923 participants were included in the final analyses.

### Ethics approval and consent to participate

The study was approved by the Institutional Review Board of Samsung Medical Center in compliance with the Declaration of Helsinki (IRB No. 2021-01-052, December 31, 2020, title: “Investigation of Evidence-Based Optimal Management Strategies for Continuous Renal Replacement Therapy”). The study procedures were followed in accordance with the ethical standards of the responsible institutional committee on human experimentation and with the Helsinki Declaration of 1975. The Institutional Review Board of Samsung Medical Center waived the requirement for informed consent due to the retrospective nature of the study and deidentified data collection^13^.

### Exposure

Anemia was defined as the requirement for RBC transfusion or the prescription of ESAs. ESA prescription was defined as having at least one prescription of darbepoetin or methoxy polyethylene glycol-epoetin, or a minimum of three prescriptions of short-acting erythropoietin during hospitalization.

### Study variables

The KNHIS records for inpatient and outpatient visits, prescriptions, and procedures were coded using the International Classification of Diseases (ICD), 10th Revision^46^. These data are highly reliable due to routine audits by the KNHIS and have been used in numerous peer-reviewed publications^47,48^. In a validation study, the diagnostic accuracy for MI in KNHIS data was reported to be 93%^49^.

We collected claim codes that encompassed information regarding comorbidities, management procedures during ICU admission, prescriptions, and demographic characteristics. Comorbidities, such as chronic liver disease, diabetes mellitus, chronic kidney disease, and cancer diagnosed within the year preceding hospitalization, were summarized using the Charlson index^50,51^. Additionally, we included in our analysis hypertension (ICD-10 codes: I10, I13, I15) and septic shock (defined as ≥ 2 days of vasopressor and ≥ 1 week of antibiotics administration), despite not being covered by the Charlson index. Management procedures included CRRT (Korean NHI procedure codes O7031-O7035, O7051-O7055) and mechanical ventilation (Korean NHI procedure codes M5857, M5858, or M5860).

### Outcomes

The primary outcomes were cardiovascular events and all-cause mortality after hospital discharge. Cardiovascular events were defined as hospitalization for heart failure (ICD-10 codes I110, I130, I132, I255, I420, I425-I429, I43, I50, and I971), acute MI (ICD-10 codes I21-I23, and I252), the presence of coronary revascularization codes (percutaneous coronary intervention, Korean NHI codes M6551-M6554, M6561-M6567, and M6571-M6572; coronary artery bypass graft surgery [CABG], O1640-O1642, O1647-O1649, OA640-OA642, and OA647-OA649), or stroke (ICD-10 codes I63, I64, and G45). The data regarding all-cause mortality was obtained from death certificates collected by Statistics Korea, part of the Ministry of Strategy and Finance of South Korea^47^.

### Subgroup analysis

We conducted subgroup analysis to evaluate the association of anemia with post-discharge cardiovascular events based on various preexisting factors. Subgroup analyses were stratified by age (age < 65 vs. ≥ 65 years old), sex, and specific comorbidities, including chronic liver disease, diabetes mellitus, chronic kidney disease, cancer, septic shock, and severe respiratory failure requiring mechanical ventilation.

### Sensitivity analysis

We performed additional analysis based on different definitions of anemia, categorizing patients into three groups: those who received only RBC transfusions, those prescribed with ESA alone, and those who received both RBC transfusions and ESA prescriptions.

### Statistical analysis

We compared the control and anemia groups using the following methods: continuous variables were presented as mean and standard deviation or as median and interquartile range, and we performed comparisons using the t-test or Mann–Whitney Test. Categorical variables were presented as numbers and proportions and compared using the chi-square test or Fisher’s exact test. Person-time was calculated from the date of hospital discharge to the event date, death, or the last follow-up date. Survival curves were generated by the Kaplan–Meier product-limit method and compared using log-rank tests. We used Cox proportional hazards regression models to estimate hazard ratio (HR) with 95% confidence interval (CI) for cardiovascular events and all-cause mortality, adjusting for age, sex, tertiary hospital, comorbidities, septic shock, CRRT duration, and mechanical ventilation. The proportional hazards assumption was assessed through log–log plots survival function and Schoenfeld residuals. To account for competing risks due to mortality, a proportional sub-distribution hazards regression model was employed for the incidence of cardiovascular events, considering death as a competing event. All analyses were two-sided and *P*-values < 0.05 were considered statistically significant. Statistical analyses were performed using SAS version 9.2 (SAS Institute, Inc, Cary, NC, USA) and R software version 3.3.2 (Free Software Foundation, Inc., Boston, MA, USA).

### Supplementary Information

Supplementary Information.

## Data Availability

The data from this study cannot be freely shared by the authors as it is the property of the Korean National Health Insurance Service (KNHIS). However, the anonymized dataset underlying this article can be obtained upon reasonable request to the National Health Insurance Sharing Service (https://nhiss.nhis.or.kr/bd/ab/bdaba000eng.do).
