# Supplementary material for: Impact of anemia requiring transfusion or erythropoiesis-stimulating agents on new-onset cardiovascular events and mortality after continuous renal replacement therapy
Source: Sci Rep. 2024 Mar 19;14:6556. doi: 10.1038/s41598-024-56772-1 (PMC10951301; doi:10.1038/s41598-024-56772-1)
Supplement: Supplementary file 1 — Supplementary Information. [file 41598_2024_56772_MOESM1_ESM.pdf]

Supplementary Table 1. Characteristics of overall patients stratified by age

	Age < 65			Age ≥ 65		
	**Control (N = 1,415)**	**Anemia (N = 3,935)**	**p-value**	**Control (N = 1,013)**	**Anemia (N = 4,560)**	**p-value**
**Age, years**	48.4 ± 11.9)	50.3 ± 10.7	< 0.001	75.6 ± 6.6	76.6 ± 6.9	< 0.001
**Sex, male**	1,073 (75.8)	2,491 (63.3)	< 0.001	705 (69.6)	2,298 (50.4)	< 0.001
**Tertiary**	677 (47.8)	2,180 (55.4)	< 0.001	437 (43.1)	2,364 (51.8)	< 0.001
**Comorbidities**						
Charlson index*	1.1 (1.4)	2.0 (2.0)	< 0.001	1.4 (1.6)	2.0 (1.9)	< 0.001
Chronic liver disease	218 (15.4)	785 (19.9)	< 0.001	63 (6.2)	306 (6.7)	0.57
Diabetes mellitus	453 (32.0)	1,074 (27.3)	< 0.001	347 (34.3)	1,463 (32.1)	0.18
Chronic kidney disease	109 (7.7)	933 (23.7)	< 0.001	137 (13.5)	1,467 (32.2)	< 0.001
Cancer	65 (4.6)	608 (15.5)	< 0.001	101 (10.0)	780 (17.1)	< 0.001
Hypertension	263 (18.6)	715 (18.2)	0.73	294 (29.0)	1,326 (29.1)	0.97
**Septic shock**	506 (35.8)	2,190 (55.7)	< 0.001	455 (44.9)	2,607 (57.2)	< 0.001
**Treatment**						
CRRT duration, days	4.2 ± 1.5	6.7 ± 4.7	< 0.001	4.2 ± 1.5	6.3 ± 4.3	< 0.001
Mechanical ventilation	471 (33.3)	1,972 (50.1)	< 0.001	304 (30.0)	1,971 (43.2)	< 0.001

Continuous variables and categorical variables are presented as means ± standard deviations and numbers (percentages), respectively.

*Charlson comorbidity was defined as the presence of disease within 1 year prior to admission.

CRRT = continuous renal replacement therapy
